# 1137. Effect of an Antibiotic Stewardship Program on Antibiotic Choice, Dosing, and Duration in Pediatric Urgent Care

**DOI:** 10.1093/ofid/ofab466.1330

**Published:** 2021-12-04

**Authors:** Amanda Nedved, Brian R Lee, Megan Hamner, Alaina Burns, Rana E El Feghaly

**Affiliations:** 1 Children’s Mercy Kansas City, Lenexa, KS; 2 Children’s Mercy Hospital, Kansas City, Missouri

## Abstract

**Background:**

Many studies have focused on decreasing inappropriate antibiotic prescriptions. In August 2018, our institution implemented an outpatient antibiotic stewardship program (ASP). We describe the impact of an outpatient ASP on the antibiotic choice, dose, and duration for common pediatric infections in a pediatric urgent care (PUC) setting.

**Methods:**

We reviewed all encounters at 4 freestanding PUC centers within our organization of patients >60 days and < 18 years with a discharge diagnosis of acute otitis media (AOM), group A streptococcal (GAS) pharyngitis, community acquired pneumonia (CAP), urinary tract infection (UTI), cellulitis, abscess, and animal bite who received systemic antibiotics between July 2017 and December 2020. We excluded patients who were transferred, admitted, or had a concomitant diagnosis that required systemic antibiotics. We used established national guidelines to determine appropriateness of antibiotic choice, dose, and duration for each diagnosis (Table 1). Our outpatient ASP efforts included the development of an antibiotic handbook, data sharing, education, quality improvement projects, and commitment letters. Pearson’s chi-square test was used to compare appropriate prescribing (choice, dose, and duration) between pre-implementation (July 2017 – July 2018) and post-implementation (August 2018 -forward). Monthly run charts evaluated improvement over time.

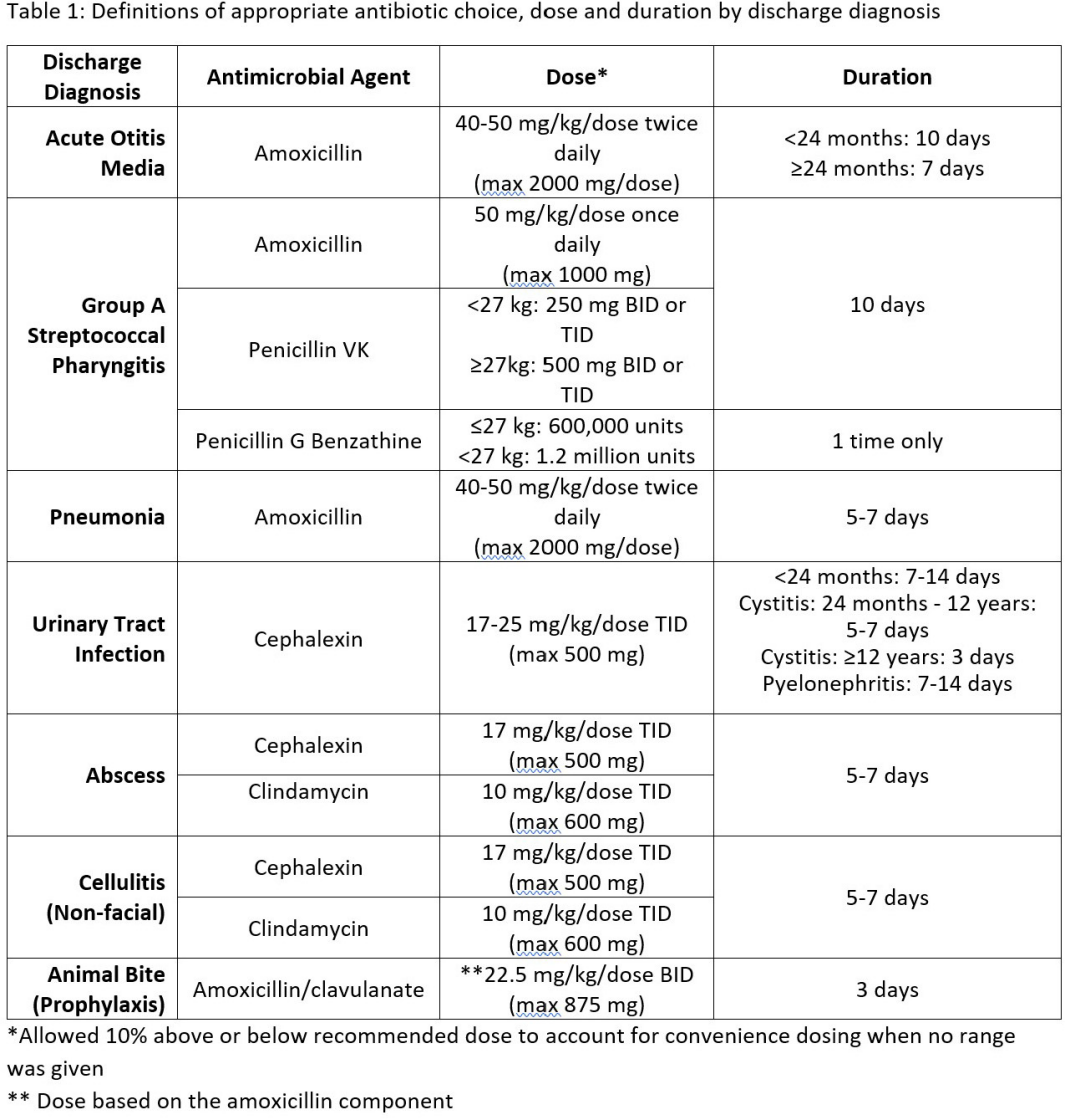

**Results:**

We included 35,915 encounters. Appropriate antibiotic agent improved in AOM (75.8% to 77.2%; p=0.03), UTI (74.9% to 89.5%; p< 0.001), cellulitis (70.5% to 75.1%; p=0.02) and abscess (53.6% to 67.7%; p< 0.001) following implementation of our ASP (Figure 1). Excluding GAS pharyngitis, all diagnoses had improvement in appropriate duration (p< 0.001) (Figure 2). Appropriate dosing improved for AOM (75.7% to 81.6%; p< 0.001), UTI (34.9% to 42.9%; p=0.01) animal bites (37.1% to 45.6%; p=0.048), and cellulitis (28.0% to 42.3%; p< 0.001) (Figure 3).

Figure 1. Appropriate Agent

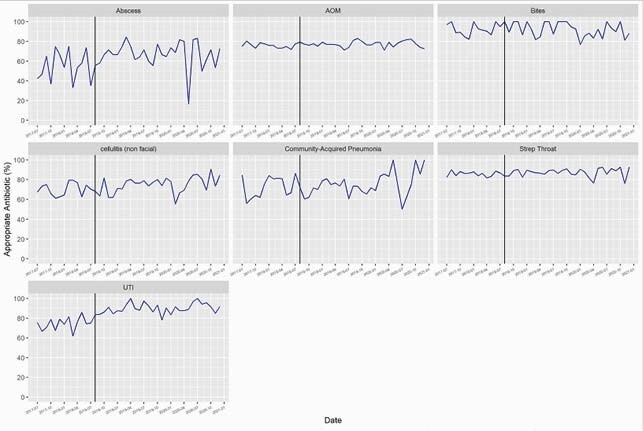

Run chart of percentage of encounters with antibiotic choice consistent with national guideline recommendations by discharge diagnosis. The vertical line indicates the start of outpatient antibiotic stewardship efforts in August 2018.

Figure 2. Appropriate Duration

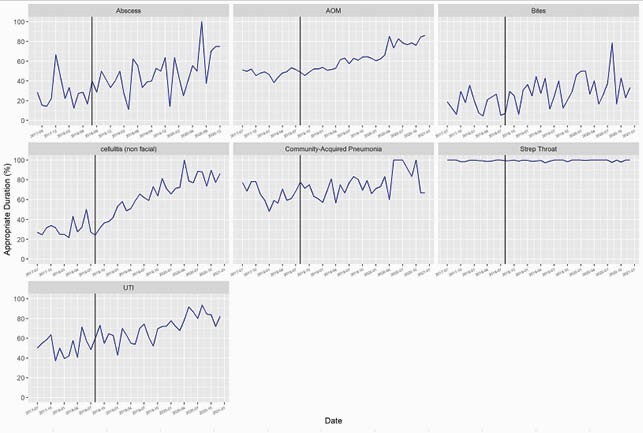

Run chart of percentage of encounters with antibiotic duration consistent with national guideline recommendations. The vertical line indicates the start of outpatient antibiotic stewardship efforts in August 2018.

Figure 3. Appropriate Dose

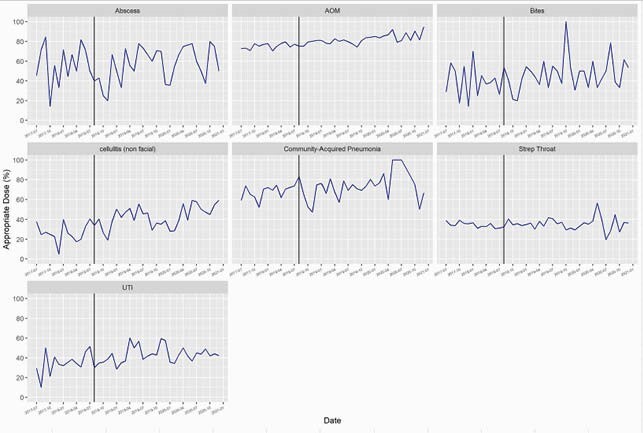

Run chart of percentage of encounters with antibiotic dose consistent with national guideline recommendations. The vertical line indicates the start of outpatient antibiotic stewardship efforts in August 2018.

**Conclusion:**

Our outpatient ASP improved prescribing patterns for agent, duration, and dose for many common pediatric infections in the PUC setting. Future work will focus on identifying opportunities to improve prescribing practices when antibiotics are indicated.

**Disclosures:**

**Brian R. Lee, PhD, MPH** , **Merck** (Grant/Research Support)**Pfizer** (Grant/Research Support)

